# Characterization of Complexes between Imidacloprid and β-Cyclodextrin: Evaluation of the Toxic Activity in Algae and Rotifers

**DOI:** 10.3390/molecules28073049

**Published:** 2023-03-29

**Authors:** Margherita Lavorgna, Martina Dragone, Chiara Russo, Gianluca D’Abrosca, Roberta Nugnes, Elena Orlo, Maria della Valle, Carla Isernia, Gaetano Malgieri, Rosa Iacovino, Marina Isidori

**Affiliations:** Department of Environmental, Biological and Pharmaceutical Sciences and Technologies, University of Campania “Luigi Vanvitelli”, Via Antonio Vivaldi 43, 81100 Caserta, Italy

**Keywords:** Imidacloprid, inclusion complex, β-cyclodextrin, toxic activity, algae rotifers

## Abstract

The development of new formulations can be driven by the knowledge of host–guest complexes using cyclodextrins which have the ability to include guest molecules within their hydrophobic cavities, improving the physicochemical properties of the guest. To rationally explore new pesticide formulations, the effects of cyclodextrins on the properties of such guest molecules need to be explored. Imidacloprid is a neonicotinoid systemic insecticide used worldwide. In this study, the inclusion complexes of Imidacloprid (IMI) with β-cyclodextrin (β-CD) were prepared in the solid state by co-precipitation and the physical mixing method, with a stoichiometry of 1:1 and 1:2 molar ratios. The obtained products, Imidacloprid:β-cyclodextrin inclusion complex (IMI:β-CD), were characterized in the solid state by Fourier transform-infrared (FT-IR) spectroscopy and X-ray powder diffractometry (XRD). In solution, the 1:1 stoichiometry for the inclusion complexes was established by the Job plot method, and the binding constant of IMI:β-CD was determined by UV–vis titration. The toxicity was determined in producers and primary consumers of the freshwater trophic chain, the green alga *Raphidocelis subcapitata* and the rotifer *Brachionus calyciflorus*, respectively. The results indicated that Imidacloprid forms inclusion complexes with CDs showing improved physicochemical properties compared to free Imidacloprid. The formation of the inclusion complex reduced the chronic toxicity in rotifers when IMI concentrations were close to those of environmental concern (tenths/hundredths of micromoles/L). Therefore, CD inclusion complexes could provide important advantages to be considered for the future industrial production of new formulations.

## 1. Introduction

Due to their role in pest management, pesticides are essential tools for crop yields of modern agriculture. In most cases, this large group of organic compounds, with various physicochemical properties, are not totally selective for the pest for which they are used. This aspect poses serious concern regarding environmental pollution and adverse effects in non-target organisms. Thus, the achievement of the balance between crop production, ecosystems and human health requires the search for new and more selective pesticide formulations with improved efficiency. The development of new ecofriendly pesticide formulations for sustainable agriculture and an overall reduction in ecotoxicity needs simple preparation processes, the use of biodegradable, biocompatible adjuvants and an improved release of active ingredients [1,2].

**Figure 1:**
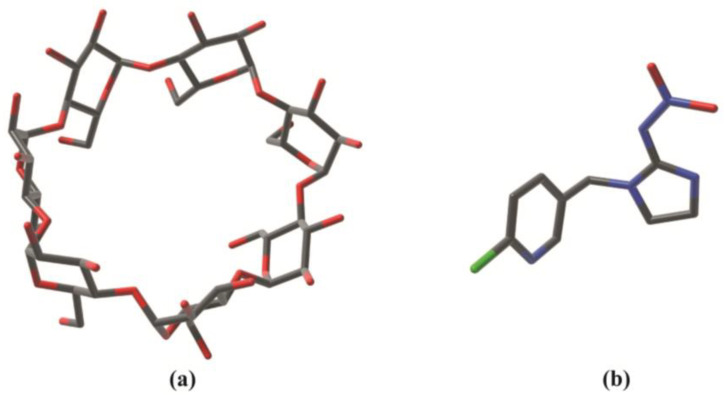
Three-dimensional structure of: (**a**) β-Cyclodextrin, (**b**) Imidacloprid. Nitrogen atoms are depicted in blue, oxygen in red, carbon in black, and chlorine in green.

Within this frame, cyclodextrins (CDs) are gaining consideration for their capacity to modify pesticide properties. Obtained from the degradation of starch by glucanotransferase, α-CD, β-CD, and γ-CD with six, seven and eight glucopyranose units, respectively, are natural oligosaccharides with a trunked cone shape that bear a low polarity cavity of different diameters [3,4] (Figure 1a). In an aqueous solution, energetically disfavored water molecules occupy this cavity and can be easily substituted by guest molecules forming host–guest inclusion complexes in a dynamic equilibrium which modifies some features of the guest molecule. The use of CDs is widely spreading in pharmacology [5], food and cosmetic fields as well as in industrial and agricultural applications [6,7,8,9,10,11]. The CD complexation of agrochemicals usually increases solubility, dissolution rate and wettability, prevents degradation by improving physical and chemical stability, enhances bioavailability and allows to overcome problems of organic solvent and surfactant use, for the extraction processes of pesticides [12,13].

CDs are not connected with side effects on humans’ health [14] and are excellent low cost tools for the complexation of many synthetic pesticides.

The use of CDs as an encapsulating device clearly depends upon the specific pesticide structure [15,16,17,18]. The inclusion can result in improved efficiency and/or the controlled release properties of the considered pesticide, thus allowing to lower the amount used with an overall positive environmental impact.

In addition to the constitution of inclusion complexes, often the mere physical mixture with CDs or the formation of external non-inclusion complexes has been shown to improve the agrochemical properties of pesticides [19]. For these reasons, to limit the negative environmental effects and to increase the agricultural benefits, insecticides may be bound to form inclusion complexes with CDs [20]. Several articles and reviews focus on different pesticide remediation strategies [21,22,23]. However, the use of these hosts in pesticide formulations and the reported studies are still limited and generally referred to the preparations and characterization of the complexes rather than being addressed to the environmental and undesirable effects of the guest molecules. In light of these considerations, the characterization of CDs’ interaction with different pesticides along with the comparative study of the toxicity of the obtained compounds is highly needed. Within this frame, we here report a physicochemical characterization and toxicological evaluation of the interaction between β-CD and Imidacloprid (IMI), a commonly used neonicotinoid systemic insecticide. 

Imidacloprid, (1-[(6-chloro-3-pyridinyl)-methyl]-N-nitro-2-imidazolidinimine (Figure 1b), is extensively used worldwide in agriculture, seed treatment, sport fields and turfs. It shows a selective toxicity towards insects compared to vertebrates acting on Na^+^ entrance and K^+^ exit blocking the neuronal transmission and determining the death of the insects [24,25]. Although the agricultural and non-agricultural advantages in the use of IMI are undoubted, its toxic effects on beneficial insects have limited its use in some countries. Furthermore, the environmental problems are increased by the strong runoff to which this insecticide is subjected due to its moderate water solubility (610 mg/L, 20 °C), low soil adsorption (log Koc 6719) and low octanol-water partition coefficient (log Kow 0.57). The resulting pollution of surface and ground waters may have harmful effects in non-target invertebrates, vertebrates and, potentially, in humans [26,27,28,29].

Considering its environmental risk, IMI inclusion complexes with β-CD are here characterized in solution by means of UV–vis and in solid state by means of X-ray Powder Diffraction and FT-IR along with molecular docking simulations. In addition, IMI toxicity in non-target aquatic organisms of two trophic levels is compared with that of the obtained complexes. The acute and chronic toxicity of each compound have been tested in the rotifer of the freshwater aquatic chain *Brachionus calyciflorus*, while the chronic toxicity in the green alga *Raphidocelis subcapitata*. Since Delogu et al., 2019 and Chen et al., 2013 reported the enhancing of insecticide activity [19,30], in this work, we also investigate the impact that the use of this insecticide with β-CD could have on the environment, for example, in waste water.

## 2. Results

To simplify the discussion, the following abbreviations will be used throughout the text: IMI for Imidacloprid, PM for physical mixture product, CP (1:1) for the co-precipitation product with a 1:1 Imidacloprid:β-CD stoichiometry and CP (1:2) for the co-precipitation product with 1:2 Imidacloprid:β-CD stoichiometry.

### 2.1. Characterization of the IMI Inclusion Complex in Solution

Changes in the guest physicochemical properties usually accompany its inclusion in the hydrophobic cyclodextrinic cavity, and this generally results in modifications of the UV–vis absorbance. This behavior is directly related to the complex concentration and can be profitably exploited for the study of the formation of inclusion complexes. IMI:β-CD stoichiometry was determined using the so-called continuous variation method or Job method: the UV–vis absorbance for a series of samples in which we have continuously varied molar ratios (R) of IMI, and β-CD has been measured. The stoichiometry is thus given by the R that corresponds to the maximum concentration of the complex. 

**Figure 2:**
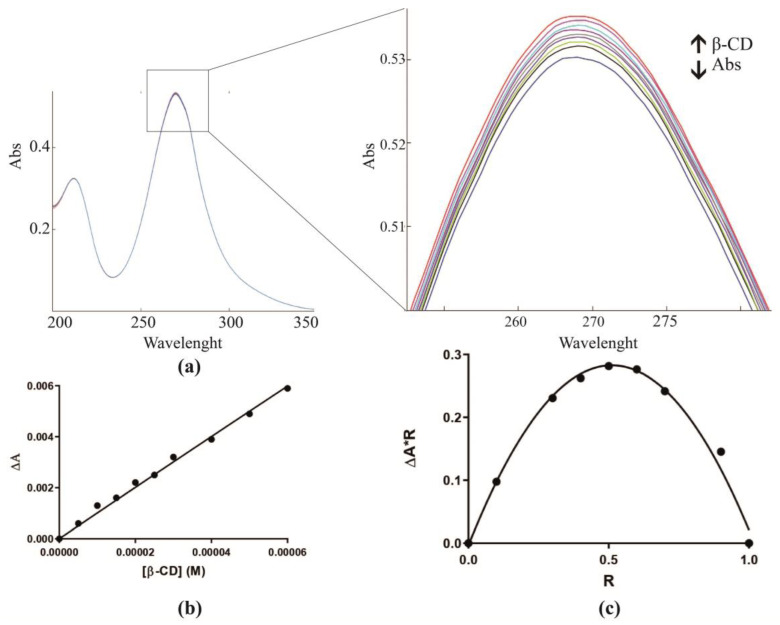
UV–vis characterization: dependence of IMI absorbance upon the addition of increasing amounts of β-CD at pH 7.1 (**a**). The binding constant of β-CD with IMI was determined by fitting the data (R^2^ = 0.9936) according to the equation reported in Materials and Methods (**b**). Job plot for the complex IMI:β-CD (λ = 270.0 nm) (**c**).

The maximum of the curve was obtained for IMI:β-CD at R = 0.5, indicating that the insecticide is included by the β-CD in a 1:1 stoichiometry (Figure 2c). 

The Job’s method allows for only the characterization of the complex stoichiometry, while the binding constant (K_b_) was estimated by monitoring the changes of the UV–vis band directly correlated with the concentration of the guest upon the gradual addition of the host. Figure 2a reports the dependence of IMI absorbance upon β-CD addition.

The maximum absorption wavelength, found at 270.0 nm, was monitored and data were fitted using a non-linear regression in order to have an accurate determination of the K_b_ that resulted in equal to 201.4 M^−1^ (Figure 2b).

When estimating the inclusion of a specific molecule in CDs, the magnitude of the K_b_ represents an important value. It is puzzling to determine which is an optimum value for a K_b_ as it basically depends on the final use of the obtained complex. High values can lead to the formation of complexes that are hard to dissociate and that can inhibit an insecticide action, although the same value could be advantageous in modifying the insecticide half-life, extending its action. The value estimated here for the IMI:β-CD complex is below the upper limit of 10,000 M^−1^, a limit indicated for CD–drug complexes in pharmaceutical applications. In agrochemical applications, this allows for us to hypothesize a ready release for the included IMI from the complexes prepared.

### 2.2. Characterization of Inclusion Complexes in Solid State 

**Figure 3:**
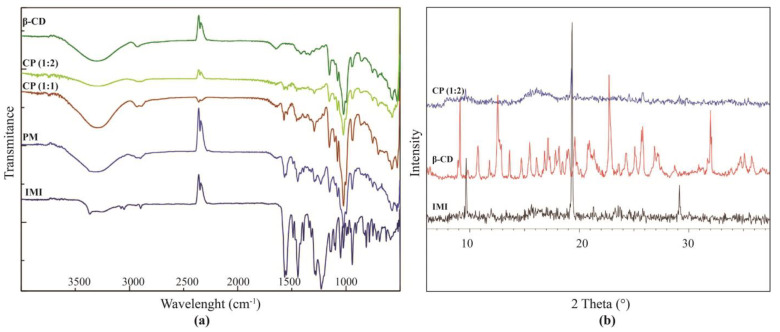
(**a**) FT-IR spectra recorded for the co-precipitation complexes of IMI with β-CD are compared to the same spectra recorded for the PM of IMI with β-CD and for IMI or the β-CD alone. (**b**) Powder XRD patterns of IMI in black, the CP in blue (1:2), and the β-CD in red.

The FT-IR spectrum of the IMI:β-CD co-precipitation product with both stoichiometries, 1:1 [CP (1:1)] and 1:2 [CP (1:2)], was compared to the spectrum of the physical mixture (PM) and to that of the pure pesticide (Figure 3a). 

In the IMI spectrum, the signals of the insecticide are readily recognizable: the peak at 3365 nm arises from the N-H stretching vibrations, those at 1552 nm and 1444 nm are characteristic of C=C aromatic bonds, while the signals at 1565 nm and 942 nm correspond, respectively, to the NO_2_ group and to the C-Cl bond [31]. The characteristic bands of β-CD are at 3290 nm for the OH stretching, at 2927 nm for the CH_2_ and at 1023 for the C-O-C bonds. The spectrum of the physical mixture is the simple sum of the spectra of both the insecticide and β-CD, indicating the lack of inclusion.

In the spectrum of the CP (1:1), there is a shift in the characteristic peaks of IMI, which become 1571 nm for the NO_2_ vibration signal, and 1542 nm and 1453 nm for the C=C aromatics, with a notable reduction in intensity that indicates the formation of an inclusion complex.

In the spectrum of the CP (1:2), the characteristic bands of the insecticide almost completely disappear, confirming the total inclusion of the molecule when in the presence of two β-CDs [32]. In this case, the interaction involves both the ring and the portion containing the NO_2_ bound to the imidazoline.

The formation of IMI:β-CD complexes by co-precipitation methods has been investigated also by means of X-ray Powder Diffraction (Figure 3b).

The peaks observed at the 2θ values of 9.68°, 19.33° and 29.16° confirm the crystalline nature of Imidacloprid. The powder diffractogram for the β-CD presents diffraction angles of 2θ at 9.08°, 10.71°, 12.64°, 14.72°, 17.11°, 21.09°, 22.80°, 24.23° and 31.96°, which are indicative of its crystalline character. In the CP (1:2), the characteristic peaks of cyclodextrin are not visible, resulting in a different crystalline state with respect to the complex; in fact, only one peak is visible. The cyclodextrin loses crystallinity due to the inclusion of the guest, and the peak at 19.33°, even if still present, is considerably reduced in intensity.

### 2.3. Molecular Docking

**Figure 4:**
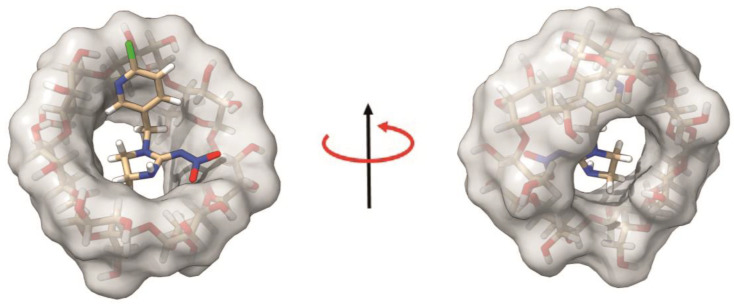
The molecular docking model of IMI in complex with the β-CD.

Molecular docking allows for the structural characterization of inclusion complexes formed by molecules with known structures [33]. For this reason, a series of molecular docking simulations were performed in order to better characterize the structural determinants of the IMI:β-CD inclusion complex. We adopted a docking protocol already used for a small organic compound bound to the β-CD [34]. The 3D structure of β-CD was downloaded from the protein data bank (PDB), while the IMI structure was found in the ChEBI (Chemical Entities of Biological Interest, https://www.ebi.ac.uk/chebi, accessed on 17 January 2023) database; both structures were protonated, energetically minimized before their use in docking simulations. The energetically favored model of the complex is reported in Figure 4. It clearly shows how the IMI is inserted with the CH_2_ group, connecting the two rings deeply inside the wider rim of the β-CD, exposing to the solvent the chloride atom bound to the aromatic ring. These data nicely reconcile with the FT-IR data which do not show any kind of shift in the band of the C-Cl bond. The interaction is mostly hydrophobic and two hydrogen bonds stabilize the complex: one made by the nitrogen of the aromatic group with an alcoholic oxygen of the sugar, and the second involving the second ring of the molecule and one of the ether oxygens of the sugar.

### 2.4. Acute Toxicity Testing 

**Figure 5:**
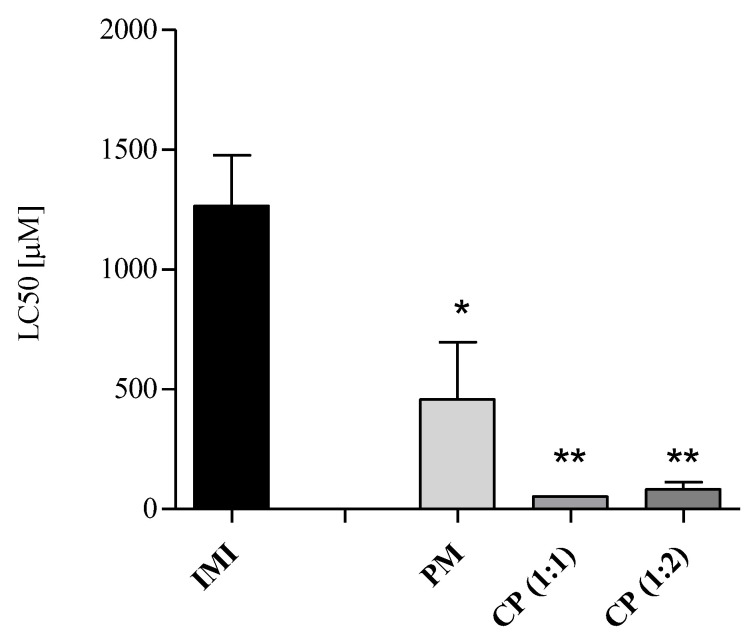
Acute toxicity in *B. calyciflorus*. LC50s expressed in µM as mean ± SD (*n* = 3). Significant differences from IMI were determined with Dunnett’s test (* *p* < 0.05, ** *p* < 0.01).

Acute toxicity results (Table 1 and Figure 5) are related to the median lethality observed in the rotifer *B. calyciflorus* in vivo exposed for 24 h to β-CD, IMI, their physical mixture (PM) and their complexes CP (1:1) and CP (1:2). LC50 values, corresponding to 50% mortality, are expressed as µM to ease the comparison among samples. β-CD showed the lowest toxicity with an effect of 16.5% at the highest concentration tested (440.54 µM). When IMI was tested alone, it induced median lethality at concentrations in the order of thousands of micromoles/L; conversely, when in a physical mixture or in complex with β-CD, the LC50s were in the order of hundreds and tens of micromoles/L, respectively (Table 1), with a statistically significant difference between IMI and PM or CPs (* *p* < 0.05, ** *p* < 0.01, Figure 5) and with a corresponding increase in toxicity. Essentially, CP (1:1) and CP (1:2) induced the highest percentage increase in toxicity (greater than 90%, Table 1) if compared to individual IMI, although no statistical difference (Tukey’s HSD multiple comparison test) was observed among PM, CP (1:1) and CP (1:2) (Figure 5). 

### 2.5. Chronic Toxicity Testing 

**Figure 6:**
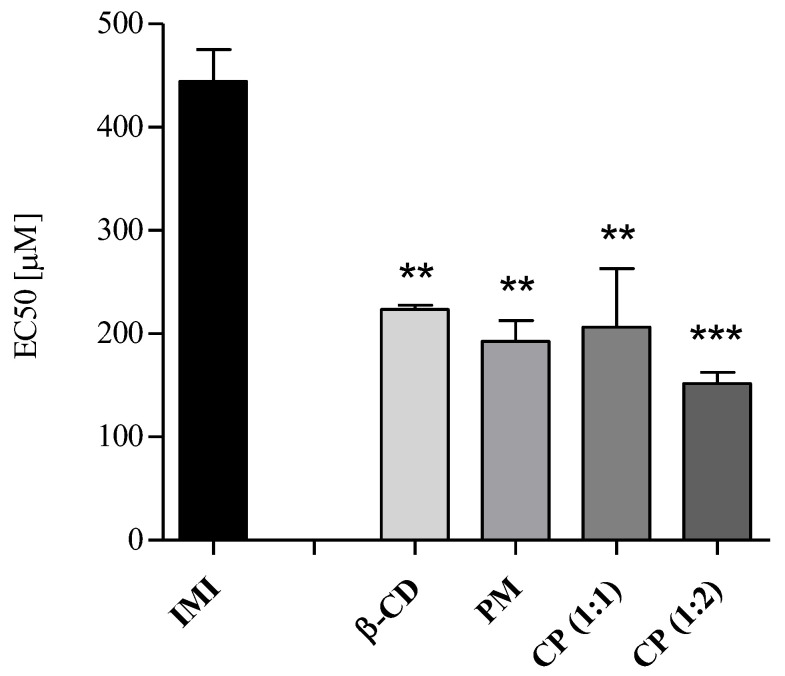
Chronic toxicity in *R. subcapitata.* EC50s expressed in µM as mean ± SD (*n* = 3). Significant differences of samples from IMI were determined for ** *p* < 0.01, *** *p* < 0.001 with Dunnett’s test.

Chronic experiments were performed using the producer *R. subcapitata* and the primary consumer *B. calyciflorus*. The results of three pooled independent experiments are presented as EC50, which corresponds to 50% inhibition of algal growth (72h-exposure) or of rotifer reproduction (48 h-exposure). As reported in Table 2, all samples induced median inhibition of algal growth at concentrations in the order of hundreds of micromoles/L. However, the comparison of IMI alone with PM and with CP (1:1) and CP (1:2) underlines an increase in chronic toxicity (greater than 50%) together with a statistically significant difference (** *p* < 0.01, *** *p* < 0.001, Figure 6), although no statistically significant difference (Tukey’s HSD multiple comparison test) was observed among PM, CP (1:1) and CP (1:2) (Figure 6). In addition, β-CD resulted in being statistically (** *p* < 0.01) more toxic than the pesticide. 

**Figure 7:**
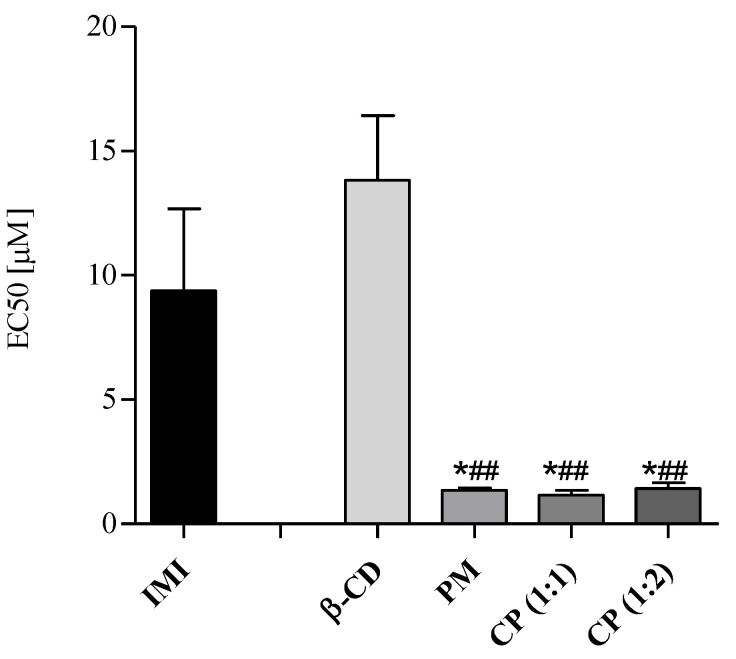
Chronic toxicity in *B. calyciflorus.* EC50s expressed in µM as mean ± SD (*n* = 3). Significant differences of samples from IMI and from β-CD were determined with Dunnett’s test and reported as * *p* < 0.05, and for ## *p*< 0.01, respectively.

Exposing B. calyciflorus to samples, EC50s (Table 3) were found at concentrations in the order of units of micromoles/L for IMI, PM and for CPs and in the order of tens for β-CD, showing higher chronic toxicity towards rotifers than algae (EC50 in the order of hundreds of micromoles/L, Table 2). However, differently from algae results, physical mixture and complexes not only resulted in being statistically more toxic than IMI but also than β-CD (Figure 7). Physical mixture and complexes determined an increase in chronic toxicity greater than 80% compared with the individual IMI (Table 3), without a statistically significant difference (Tukey’s HSD multiple comparison test) among PM, CP (1:1) and CP (1:2) (Figure 7).

**Figure 8:**
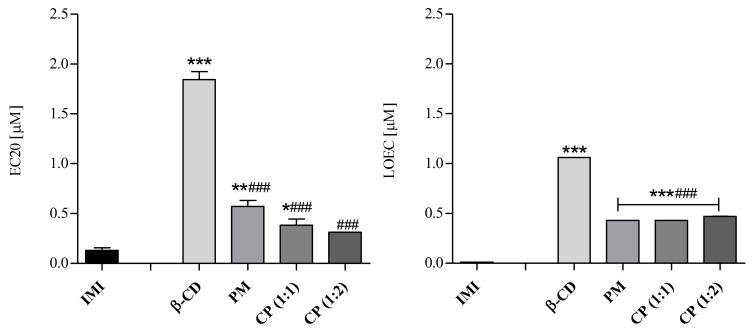
Chronic toxicity testing in *B. calyciflorus*. EC20s and LOECs expressed in µM as mean ± SD (*n* = 3). Significant differences of samples from IMI and from β-CD were determined with Dunnett’s test and reported as * *p* < 0.05, ** *p* < 0.01, *** *p* < 0.001, and as ### *p* < 0.001, respectively.

EC10, EC20, NOEC and LOEC values were estimated to understand the chronically toxic starting effects. Interestingly, the toxic effect pattern changed between producers and consumers when the tested concentrations decreased. In the case of the producer *R. subcapitata*, EC10 and EC20 as well as NOEC and LOEC values were higher for IMI than for both physical mixture and complexes, which continued to be more toxic with respect to the pesticide alone (Table 4), while for the consumer *B. calyciflorus*, the results appeared to be reversed with a statistical reduction in IMI toxicity when in a physical mixture or in complexes with β-CD (Table 4, Figure 8).

Basically, considering EC20 IMI value, the inhibition of reproduction of B. calyciflorus decreased of 81% for PM and of 69 and 66% for CP (1:1) and CP (1:2), respectively. Furthermore, the LOEC values (Table 4), slightly differing from EC20s because of their dependence from concentrations chosen by the operator, highlighted a reduction of 98% of IMI toxicity when it was mixed or complexed with β-CD. This reduction is statistically appreciable (Figure 8).

## 3. Discussion

Interactions with CDs can change the physicochemical properties of a guest molecule, leading to improvements in terms of solubility and bioavailability. The new features can be exploited to obtain novel formulations of pesticides with better efficacies or release properties that allow for us to spread a lower quantity of pesticides, thus reducing their environmental impact. For these motives, we have here reported the study of the interaction between β-CD and IMI, whose environmental risk quotient worldwide was calculated by Nugnes and collaborators [29]. They have shown how this quotient can reach, in some cases, values above the threshold of 1, which is considered to be of high environmental concern. The structural analysis has been performed in solution and in solid state using complementary techniques. The stoichiometry of the complexes in solution was established using the Job plot method to be 1:1, and the binding constant estimated by means of UV–vis spectroscopy was 201.4 M^−1^. The molecular docking studies have demonstrated how the β-CD toroidal shape well accommodates IMI deeply inside the wider rim of the β-CD. The formation of the 1:1 and 1:2 IMI:β-CD inclusion complexes in the solid state was confirmed by X-ray powder diffractometry and FT-IR spectroscopy. We then compared the toxicity of the obtained complexes to that of the IMI alone. This latter was proven to be acutely toxic towards the primary consumer *B. calyciflorus*, inducing 50% mortality at high concentrations, equal to 1234.83 µM (24 h-exposure), in line with the results previously obtained by Nugnes and collaborators [29] who reported a LC50 higher than 1173 µM in the same non-target freshwater organism. When IMI was in a physical mixture, the median mortality increased by 67%, while in complexes with β-CD, the mortality increased by percentages greater than 90%. CDs are known to act as permeation promoters, increasing the availability of the guest on the surface of biological barrier, particularly via chlolesterol pharmacokinetics [35,36,37]. The enhancement in penetration does not seem to be linked to complexation, which is instead important when a drug-carrying function is required [19]. 

This research turns out to be one of the first pioneering studies that aims to evaluate the effects of mixtures and complexes of IMI:β-CD in freshwater non-target organisms.

Our acute data demonstrate that the inclusion of IMI by β-CD, as explained by Morillo González et al., 2006 [38], enhance the toxicity of this pesticide on non-target organisms. 

Considering that the IMI acute EC50 value was definitely above the concentrations of environmental concern, going from hundredths of nanomoles/L to tenths of micromoles/L [26,28,39,40,41], and that the aquatic organisms are exposed to IMI residues for a prolonged period of time, we decided also to evaluate the long-term toxicity. 

The IMI chronic toxic effect was more evident towards consumers (48 h exposure) than producers (72 h exposure) with EC50 values at concentrations equal to 8.99 µM and 442.38 µM, respectively, in line with results obtained by Nugnes and colleagues [29] who chronically tested IMI in algae and rotifers. The higher sensitivity to IMI shown by the rotifers, multicellular organisms, compared to the algae, unicellular organisms, could be due to the inhibition of the excitatory postsynaptic potential exerted by the pesticide against multicellular organisms [42]. However, a statistically significant increase in the EC50s of IMI was observed in both organisms when the pesticide was in mixture or in complex with β-CD; in particular, when exposing rotifers to PM and CPs, the increase in EC50s was greater than 80%, suggesting that CDs enhance the penetration of the free pesticide fraction. The toxicity of the individual β-CD found in algae cannot be underestimated, and could be due to alterations in membrane components as cholesterol and phospholipids that lead to algal cytotoxicity, as explained by Fai and collaborators [43]. However, the low chronic toxicity of β-CD detected in rotifers is confirmed by the study of Adriaens and collaborators [44]. Interestingly, in consumers, when tested concentrations decreased to values below those of environmental concern (tenths/hundredths of micromoles/L), a statistical reduction in IMI toxicity was observable up to 98% when it was in physical mixture or in complexes with β-CD. Thus, our results indicate that the inclusion of the IMI in β-CD could be advantageous from an environmental point of view, determining a lower toxicity towards non-target organisms compared to the individual IMI and allowing for a more rational use of the pesticide.

## 4. Materials and Methods

### 4.1. Materials

Imidacloprid (CAS: 138261-41-3) was purchased from Sigma Aldrich (Milano, Italy). A total of 0.1 mM of the solutions of IMI was produced by dissolving the appropriate amount of pesticide in 1.0 mM of the phosphate-buffered solution. The pH was kept constant at 7.1 and continuously checked through a calibrated CRISON pH-meter Basic 20. Solvents and reagents of analytical grade, double-distilled-MilliQ water were used.

### 4.2. IMI:β-CD Binding Constant by Means of UV–VIS Spectroscopy

The molar ratio titration method was used to estimate the binding constant for the complexes produced. A 0.05 mM buffered solution of IMI was gradually titrated with a β-CD solution at different concentrations ranging from 0 to 0.15 mM. Analytical differences between the free IMI and its complex form allowed for us to successfully estimate K_b_ by means of direct spectroscopic methods. Hence, changes in IMI absorption intensity were examined as a function of β-CD concentrations; the maximum absorption wavelength for IMI was recorded at 275.0 nm. To appropriately calculate the K_b_, we reordered the Benesi–Hildebrand equation into [45]:

(1) $$\Delta A=\frac{\left[\beta -\mathrm{CD}\right]\cdot K_{b}\cdot \;\varepsilon \cdot \left[\mathrm{pesticide}\right]}{1+K_{b}\cdot \left[\beta -\mathrm{CD}\right]}$$

where ΔA is the absorbance difference of IMI in the absence and presence of β-CD. K_b_ was obtained from the titration curve data ΔA as a function of β-CD concentration fitted by non-linear regression using the Graphpad software.

### 4.3. IMI:β-CD Complex Stoichiometry Determination by Means of the Job Plot Method

Stoichiometry for the IMI/β-CD complex was determined by means of the continuous variation method (Job method) [46]. Concisely, 0.01 mM of the un-buffered solutions of IMI and of β-CD were mixed at different molar ratios $R=\frac{\left[\mathrm{pesticide}\right]}{\left[\mathrm{pesticide}\right]+\left[\beta -\mathrm{CD}\right]}$, keeping the final volume constant. The stoichiometric ratio of the complex was found as the maximum R of the curve that plots (ΔA × R) against R; ΔA is the difference in absorbance of IMI in the absence and presence of β-CD. The experiments were performed at room temperature in a 1 cm quartz cuvette and data were acquired in the 200–400 nm wavelength range with a Shimadzu UV-1800 spectrometer.

### 4.4. Preparation of IMI:β-CD Inclusion Complexes in Solid State

The co-precipitation method was used to prepare the solid-phase inclusion complexes [47]. A saturated solution of β-CD was prepared and heated to 60° for half an hour while stirring. The insecticide dissolved in 200 µL of methanol was added to this solution and stirred for two hours. For the IMI:β-CD complex, a 1:1 molar ratio of 23.0 mg of IMI and 100 mg of β-CD were used; for the IMI:β-CD complex with a 1:2 molar ratio, were weighed 11.5 mg of insecticide and 100 mg of β-CD. Once a homogeneous solution was reached, this was left at room temperature overnight to obtain a precipitate that was then filtered and washed with chloroform and methanol. After the washing procedure, samples were dried in an oven at 60 °C.

Physical mixture was obtained by blending pesticides and β-CD powders in an agate mortar to gain a homogeneous mixture with 1:1 molar ratio.

### 4.5. X-ray Powder Diffraction (XRD)

Diffraction powder patterns were collected at room temperature and 40 kV and 30 mA on a Bruker D8 Advance diffractometer with a graphite monochromator and a tube anode Cu (λ = 1.54). The diffractograms were acquired in the 2θ angle range of 6–40° and process parameters with the scanning speed 0.04 θ/s.

### 4.6. Fourier Transform Infrared (FT-IR) Spectroscopy

The FT-IR spectra were recorded on a Jasco FT-IR 4100 spectrometer. Samples were obtained into a fine powder using a mortar. The characteristic peaks of IR transmission spectra were recorded at a resolution of 0.4 cm^−1^ over a wavenumber region of 400–4000 cm^−1^.

### 4.7. Molecular Docking Studies

For the molecular docking studies of the IMI:β-CD inclusion complex, the package Hex version 3.1.0 was used for the software Samson version 2022 R2. The PDB files of the receptor (β-CD) and the ligand (IMI) were the Hex inputs, and the parameters used are reported in [48]. Structure refinement and the energy minimization of the complex were also accomplished with Hex. Computations were performed by means of the shape complementary scoring function, with 16 and 30 expansion orders for the initial and final steps, respectively. In each molecular docking calculation, the CD was retained as a fixed truncated cone and the guest IMI was allowed to freely move. Molecular docking results were clustered into groups on the bases of the root mean-square deviation values and host–guest inclusion complex, with the lowest energy conformation selected and analyzed. The complexes were visualized and analyzed using the software Samson that was also used to validate all input files.

### 4.8. Toxicity Testing

Stock solutions were prepared dissolving powder in Milli-Q water. Test solutions were freshly prepared in standard synthetic media. 

#### 4.8.1. Acute Toxicity Testing

The rotifer *B. calyciflorus* was used in acute toxicity testing according to ASTM E 1440-91 (2004). 

The organisms were hatched from cysts in moderately hard synthetic freshwater (80–100 mg/L CaCO_3_, pH 7.5 ± 0.3) for 16–18 h at 25 ± 1 °C under lighting of 3000–4000 lux, and those less than 2 h old were used in the tests. Five rotifers and 0.3 mL of test solution were placed in each well in six replicates and the plates were incubated in darkness at 25 °C for 24 h. After range-finding tests, we chose concentrations starting from the highest tested equal to 500 mg/L; this was diluted with a geometric progression of 2, but to make the comparison between samples easier, the concentrations were reported in [μM], IMI: from 30.56 µM to 1955.71 µM; β-CD: from 27.53 µM to 440.54 µM; PM: from 22.47 µM to 359.55 µM; CP(1:1): from 11.23 µM to 719.09 µM; CP(1:2): 6.19 µM to 197.97 µM.

The negative control was prepared by exposing the organisms only to standard media. Mortality was the endpoint of the acute test, and the results were expressed as Median Lethal Concentration (LC50), the concentration causing the 50% effect. 

#### 4.8.2. Chronic Toxicity Testing

The producer *R. subcapitata* and the consumer *B. calyciflorus* were used in chronic toxicity testing according to OECD 201 (2011) with slight modifications, as reported by Paixao et al. [49] and ISO 20666 (2008) [50], respectively. 

The algal test was performed incubating 0.2 mL of the test solutions with 0.1 mL of algal suspension (10^4^ cells/mL) in 96-well plates. After range-finding tests, the chosen concentrations started from the highest tested which was equal to 500 mg/L; this was diluted with a geometric progression of 2. For IMI only, the highest concentration was 400 mg/L; however, as mentioned above, to make the comparison between samples easier, the concentrations were reported in [μM], IMI: from 3.05 µM to 1564.57 µM; β-CD: from 3.44 µM to 440.54 µM; PM: from 2.81 µM to 359.55 µM; CP(1:1): from 2.81 µM to 359.55 µM; CP(1:2): 3.09 µM to 197.97 µM.

The plates were incubated at 25 ± 1 °C under 6000 lx and read at 450 nm using the microplate reader (Synergy H1, Biotek, Winooski, VT, USA) immediately before the test and after 24 h, 48 h and 72 h. 

The rotifers were hatched as described above and those less than 2 h old were used in the tests. A total of 0.9 mL of the test solutions and 0.1 mL of fresh suspension of 10^7^ cells/mL of the unicellular alga *R. subcapitata* with one rotifer were placed in each well in 48-well plates. Plates were incubated in darkness at 25 °C for 48 h. After range-finding tests, the chosen concentrations started from the highest tested which was equal to 10 mg/L with a geometric progression of 2; for IMI only, the highest concentration was 102.4 and the geometric progression was equal to 3.2. The concentrations in [μM] were: IMI: from 12.52 µM to 400.53 µM; β-CD: from 0.55 µM to 8.81 µM; PM: from 0.45 µM to 7.19 µM; CP(1:1): from 0.45 µM to 7.19 µM; CP(1:2): 0.25 µM to 3.96 µM.

In each test, the negative control was prepared by exposing the organisms to only standard media. Inhibition of the reproduction of the rotifers and inhibition of algal cellular growth were the endpoints of the chronic tests, and the results were expressed as the chronic Effective Concentrations (ECx). 

#### 4.8.3. Toxicity Data Analysis

For each toxicity test, three independent experiments were performed, and the effect percentages were pooled to estimate the concentrations giving x% effect by non-linear regression (log agonist vs. normalized response-variable slope, Prism5 software, Graphpad Inc., San Diego CA, USA). ANOVA and Dunnett’s multiple comparison test were used to estimate the No Observed Effect Concentration (NOEC) and the Lowest Observed Effect Concentration (LOEC) by significant differences from the negative control (* *p* < 0.05, ** *p* < 0.001 and *** *p* < 0.0001). Significant differences of samples from IMI were determined with Dunnett’s test for * *p* < 0.05, ** *p* < 0.01, *** *p* < 0.001, while significant differences from β-CD were determined for # *p* < 0.05, ## *p* < 0.01, ### *p* < 0.001. Tukey’s HSD (*p* < 0.05) was used to examine the differences among samples.

## 5. Conclusions

Cyclodextrinic complexes have been compared with the guest molecule alone and with the simple physical mixtures in which the molecule is not included in the CD cavity. Physical mixtures of CD and organic molecules have rarely produced effects comparable with inclusion complexes regarding stability, solubility, and other chemical parameters; however, very little is known about the enhancement of the bioavailability.

Our findings indicate how the interaction of IMI with β-CD can sensitively lower the toxicity of the insecticide when used in concentrations close to those of environmental concern, leading to an overall beneficial environmental impact. This opens new scenarios for the reduction in IMI residues in the environment and a new approach for the development of future pesticide remediation processes from soil and water using CDs as encapsulating agents.

The use of effective and safe physical mixtures instead of producing a complex could be also of considerable economic and practical impact in the development and sustainable use of pesticides.

## Figures and Tables

**Table 1:** Acute toxicity in *B. calyciflorus*. LC50 values (95% confidence intervals) are expressed in µM, the increase in the median toxicity of PM and/or CPs vs. IMI is expressed in percentage.

	β-CD	IMI	PM	CP (1:1)	CP (1:2)
LC50 [µM]	n.d.*	1234.83	404.56	53.69	76.89
		(1034.18–1474.21)	(244.63–668.90)	(43.00–67.04)	(56.62–104.37)
Acute toxicity Increase in PM/CP vs. IMI	-	-	67.23	95.65	93.77

n.d.*: 16.5% effect at 440.54 µM.

**Table 2:** Chronic toxicity in *R. subcapitata*. EC50 values (95% confidence intervals) are expressed in µM, the increase in the median toxicity of PM and/or CPs vs. IMI is expressed in percentage.

*R. subcapitata*	β-CD	IMI	PM	CP (1:1)	CP (1:2)
EC50 [µM]	223.79	442.38	193.08	199.12	150.54
	(181.32–276.21)	(365.33–535.86)	(135.40–275.20)	(140.80–281.52)	(89.76–252.45)
Chronic toxicity Increase in PM/CP vs. IMI	-	-	56.35	54.99	65.97

**Table 3:** Chronic toxicity testing in *B. calyciflorus*. EC50 values (95% confidence intervals) are expressed in µM, the increase in the median toxicity of PM and/or CPs vs. IMI is expressed in percentage.

*B. calyciflorus*	β-CD	IMI	PM	CP (1:1)	CP (1:2)
EC50	13.57	8.99	1.37	1.15	1.42
	(10.84–17.00)	(5.87–13.30)	(1.15–1.58)	(0.93–1.44)	(1.11–1.82)
Chronic toxicity Increase in PM/CP vs. IMI	-	-	84.74	87.21	84.20

**Table 4:** Chronic toxicity testing in *R. subcapitata* and *B. calyciflorus*. EC10, EC20, NOEC and LOEC values (95% confidence intervals) are expressed in µM.

	β-CD		IMI		PM		CP (1:1)		CP (1:2)	
[µM]	*R. s.*	*B. c.*	*R. s.*	*B. c.*	*R. s.*	*B. c.*	*R. s.*	*B. c.*	*R. s.*	*B. c.*
EC20	37.18	1.85	128.69	0.11	24.16	0.57	24.88	0.36	18.49	0.32
	(27.4–48.46)	(1.41–2.29)	(93.48–170.54)	(0.06–0.24)	(14.61–36.62)	(0.43–0.72)	(15.31–37.10)	(0.29–0.50)	(9.63–30.45)	(0.20–0.43)
EC10	13.04	0.53	62.19	0.01	7.19	0.36	7.33	0.21	5.42	0.12
	(8.37–19.82)	(0.35–0.79)	(41.07–95.05)	(0.004–0.03)	(3.47–14.11)	(0.21–0.43)	(3.59–14.17)	(0.14–0.29)	(2.11–12.35)	(0.08–0.23)
LOEC	13.83	1.06	12.20	0.01	11.29	0.43	11.29	0.43	3.09	0.47
NOEC	6.87	0.53	6.10	0.003	5.61	0.21	5.61	0.21	1.54	0.24

## Data Availability

Data used and/or analysed in this study are available from the corresponding author on request.

## Sample Availability

Samples of the compounds are available from the authors.
